# Biomarker Associations in Delayed Cerebral Ischemia after Aneurysmal Subarachnoid Hemorrhage

**DOI:** 10.3390/ijms23158789

**Published:** 2022-08-07

**Authors:** Dora Spantler, Tihamer Molnar, Diana Simon, Timea Berki, Andras Buki, Attila Schwarcz, Peter Csecsei

**Affiliations:** 1 Department of Anaesthesiology and Intensive Care and Department of Neurosurgery, Medical School, University of Pecs, 7624 Pecs, Hungary; 2 Department of Anaesthesiology and Intensive Care, Medical School, University of Pecs, 7624 Pecs, Hungary; 3 Department of Immunology and Biotechnology, Medical School, University of Pecs, 7624 Pecs, Hungary; 4 Department of Neurosurgery, Faculty of Medicine and Health, Örebro University, 702 81 Örebro, Sweden; 5 Department of Neurosurgery, Medical School, University of Pecs, 7624 Pecs, Hungary

**Keywords:** aneurysmal subarachnoid hemorrhage, delayed cerebral ischemia, functional outcome, MCP-3, CX3CL1, IP-10

## Abstract

The prognosis for patients with aneurysmal subarachnoid hemorrhage (aSAH) is heavily influenced by the development of delayed cerebral ischemia (DCI), but the adequate and effective therapy of DCI to this day has not been resolved. Multiplex serum biomarker studies may help to understand the pathophysiological processes underlying DCI. Samples were collected from patients with aSAH at two time points: (1) 24 h (Day 1) and (2) 5–7 days after ictus. Serum concentrations of eotaxin, FGF-2, FLT-3L, CX3CL1, Il-1b, IL-4, IP-10, MCP3, and MIP-1b were determined using a customized MILLIPLEX Human Cytokine/Chemokine/Growth Factor Panel A multiplex assay. The functional outcome was defined by the modified Rankin scale (favorable: 0–2, unfavorable: 3–6) measured on the 30th day after aSAH. One-hundred and twelve patients with aSAH were included in this study. The median level of CX3CL1 and MCP-3 measured on Days 5–7 were significantly higher in patients with DCI compared with those without DCI (CX3CL1: with DCI: 110.5 pg/mL, IQR: 82–201 vs. without DCI: 82.6, 58–119, *p* = 0.036; and MCP-3: with DCI: 22 pg/mL (0–32) vs. without DCI: 0 (0–11), *p* < 0.001). IP-10, MCP-3, and MIP-1b also showed significant associations with the functional outcome after aSAH. MCP-3 and CX3CL1 may play a role in the pathophysiology of DCI.

## 1. Introduction

Despite only accounting for 5% of all strokes, aneurysmal subarachnoid hemorrhage (aSAH) imposes a significant health burden on society due to its estimated 40% mortality rate [1]. Medical complications following aSAH such as rebleeding, hydrocephalus, cerebral vasospasm, and delayed cerebral ischemia (DCI) contribute significantly to disease long-term functional outcome. The prognosis for patients with aSAH is heavily influenced by the development of delayed cerebral ischemia, but adequate and effective therapy of DCI to this day has not been resolved [2]. For decades, the condition was attributed to cerebral vasospasm (CV); however, about 20% of SAH patients develop DCI without evidence of CV and only 30% of patients with CV actually suffer from DCI [3]. Recent evidence suggests that several factors influence the development of DCI, such as vascular dysfunction, elevation of intracranial pressure (ICP), microthrombosis, autoregulatory failure, neuroinflammation, disruption of the blood–brain barrier (BBB), cell death, oxidative stress, and cortical spreading depolarization [4]. Vasospasm is only one of them. These factors, combined with direct injury caused by initial bleeding, form the phenomenon of early brain injury (EBI) [5]. EBI is thought to play an important role in the development of DCI [6]. A consensus definition of DCI has been developed and is widely used [7]. However, the clinical diagnosis of DCI in conscious or sedated patients is particularly difficult as it is almost impossible to assess consciousness in a sedated patient [8]. In addition, the diagnosis of DCI is easier in patients who are fully conscious, but the prognostic factors affecting the effectiveness of the therapy still remain unknown. The only evidence-based strategy for the prevention and treatment of DCI is nimodipine, which can prevent and reverse spasm of small vessels but has no effect on vasospasm of larger vessels [9]. Endovascular strategies have been used for radiographic vasospasm, including balloon angioplasty and placement of intracranial stents [9]. Considering all this, a laboratory biomarker would be an ideal solution, which would enable timely, specific, and sensitive diagnosis of DCI in SAH patients. Unlike other diseases where serum biomarkers are routinely used, however, there are still no effective serum biomarkers in clinical practice for predicting DCI or monitoring therapeutic efficacy. The study of a single biomarker is not suitable for the complex characterization of the mechanisms underlying DCI, but the analysis of a comprehensive biomarker profile may be more appropriate [10]. To have a broader view of possible pathophysiological processes, we performed a multiplex serum biomarker analysis. The biomarkers measured in this study were selected according to the following criteria: (i) previously their role was investigated in animal models of ischemic stroke or SAH, such as eotaxin [11], fibroblast growth factor-2 (FGF-2) [12,13], chemokine (C-X3-C motif) ligand-1 (CX3CL1) [14], or interleukin-1b (Il-1b) [15]; (ii) its role in the case of SAH has already been investigated, but the studies mainly focused on its level in the CSF, such as monocyte chemotactic protein-3 (MCP-3) [10]; or (iii) its pathophysiological role was primarily investigated in human ischemic stroke such as Fms related receptor tyrosine kinase-3 ligand (FLT-3L) [16] and in SAH (interferon gamma-induced protein 10, IP-10) [17,18], macrophage inflammatory protein 1-beta (MIP-1b) [17], and, further, a more detailed investigation may be promising in the case of SAH. The great variability of temporal patterns of inflammation-related proteins is an indicator of the complexity of the inflammatory response following aSAH. Their exact role is not easy to establish, especially considering the fact that many of these substances are described to play both a detrimental and a beneficial role in the disease course depending on the time after bleeding [10]. We planned to investigate the relationship of the above markers to DCI and the functional outcome and their relationship to each other in the present study.

## 2. Results

### 2.1. Patients’ Characteristics

One-hundred and twelve patients with aSAH (Table 1) were included in this study. Patients were enrolled between November 2018 and December 2021. All (100%) of the aneurysms were secured by coiling. Patients hada mean age of 57 (SD13) and 62% were female. Of them, 38 patients with aSAH (34%) presented to the emergency department with a WFNS Grade I. Almost half of the patients had a history of arterial hypertension (43.8%) and 11% had a history of smoking. Nearly one-third of the patients had DCI (29.1%) during their in-hospital stay. A description of the of these aSAH patients is shown in Table 1.

### 2.2. Cytokines Associated with DCI and Functional Outcome

**Figure 1:**
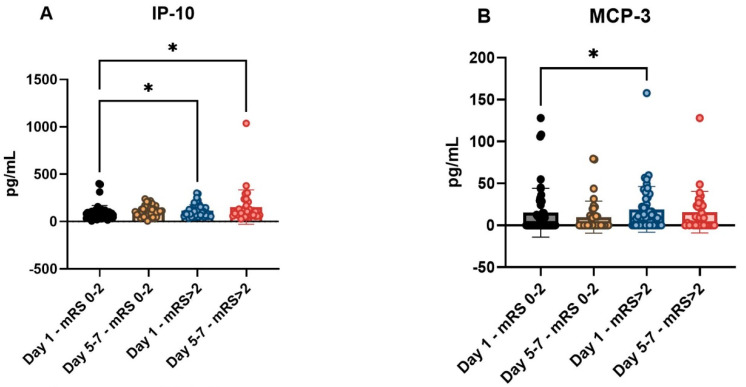

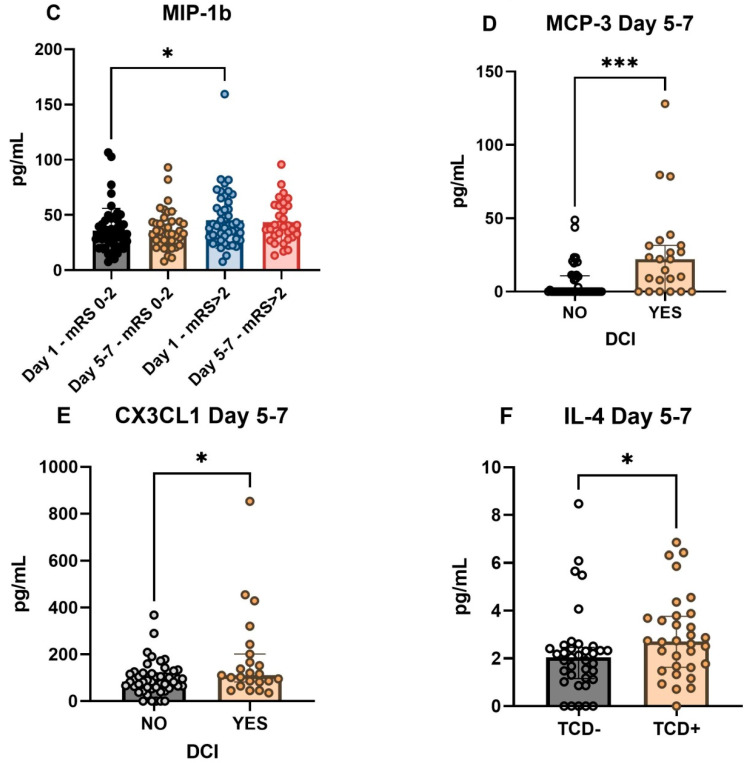
Characteristics of serum biomarker levels in different clinical subgroups in patients with aSAH. Correlation of the functional outcome with the investigated biomarkers, in the case of IP-10 (**A**), MCP-3 (**B**) and MIP-1b (**C**). Correlation of MCP-3 (**D**) and CX3CL1 (**E**) measured at T2 with DCI. Association of IL-4 (**F**) with TCD positivity. The functional outcome was examined 30 days after admission and characterized on the modified Rankin scale (mRS). Biomarker sampling times: Day 1, 24 h after aSAH, Day 5–7, 5–7 days after aSAH. DCI, delayed cerebral ischemia; aSAH, aneurysmal subarachnoid hemorrhage; TCD, transcranial Doppler ultrasound; CX3CL1, chemokine ligand 1, also known as fractalkine; IL-4, interleukin-4; IP-10, interferon gamma-induced protein 10, also known as C-X-C motif chemokine ligand 10 (CXCL10); MCP-3, Monocyte chemotactic protein-3; MIP-1b, macrophage inflammatory protein 1-beta. * denotes *p* < 0.05, *** denotes *p* < 0.001.

None of the cytokines tested on Day 1 were associated with DCI, whereas only cytokines measured on Day (IP-10, MCP-3, MIP-1b) were associated with functional outcome (Table 2). CX3CL1 and MCP-3 measured on Days 5–7 were significantly higher in patients with DCI compared to those without DCI (CX3CL1: Day 5–7, without DCI: 82.6 pg/mL, IQR: 58–119 vs. Day 5–7, with DCI: 110.5 (82–201), *p* = 0.036 and MCP-3: Day 5–7, without DCI: 0 (0–11) vs. Day 5–7, with DCI: 22 (0–32), *p* < 0.001, Figure 1). Serum IP-10 levels in patients with poor outcomes were significantly higher than in patients with favorable outcomes at both time points (Day 1, favorable outcome: 74.7 pg/mL, IQR: 43–97 vs. Day 1, unfavorable outcome: 100, 68–146, *p* = 0.005 and Day 1, favorable outcome: 74.7 pg/mL, IQR: 43–97 vs. Day 5–7, unfavorable outcome: 98.8, 65–157, *p* = 0.004). For MCP-3 and MIP-1b, the serum concentrations measured on Day 1 showed significantly higher levels in patients with unfavorable outcome compared with the group with favorable Day 30 outcome (MCP-3: Day 1, favorable: 0 pg/mL, IQR: 0–15 vs. Day 1, unfavorable: 11.8, 0–25, *p* = 0.045 and MIP-1b: Day 1, favorable: 31.8 pg/mL, 23–42 vs. Day 1, unfavorable: 40, 28–56, *p* = 0.025, Figure 1).

In order to clarify how the increase of MCP-3 and CX3CL1 seen in DCI is related to the time of DCI, we performed an additional analysis. Average time of onset of DCI in our cohort was 6 ± 3.2 days (mean ± SD). We grouped the DCI cases based on the sampling dates: the cases before T2 were in group A, while the cases after T2 were in group B, Table 3.

### 2.3. Clinical Variables Associated with DCI and Day 30 Functional Outcome

We found no significant association between admission WFNS and Fischer scores and the development of DCI in aSAH patients. Similarly, there was no association between demographic (female, age) and clinical risk factors (hypertension, diabetes, smoking) and the emergence of DCI during hospital stay (Table 4). The admission GCS score was significantly lower in the DCI group than in the non-DCI group (DCI: 9, IQR: 5–14 vs. no DCI: 14, 10–15, *p* = 0.02). Decompressive craniotomy was required more frequently in the DCI group, but there was no difference between the two groups in terms of EVD use. Other factors related to DCI and Day 30 functional outcome are shown in Table 4. We found that regardless of whether the patient had an infection or not during hospitalization, the serum level of MCP-3 was significantly higher in the DCI group than in the non-DCI group. In contrast, de CX3CL1 concentration measured at T2 did not show a significant difference in the two groups of DCI, regardless of the presence of an infection (Table 5).

### 2.4. Correlations between Biomarkers in aSAH Patients

**Figure 2:**
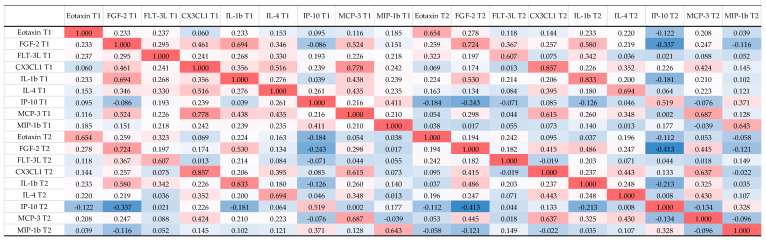
Correlation between different serum biomarkers in patients with aSAH. (Note: red indicates that the two parameters were positively correlated, and blue indicates that the two parameters were negatively correlated; the darker the color, the stronger the correlation). T1, serum sample at Day 1 after aSAH; T2, serum sample at Day 5–7 after aSAH. FGF-2, fibroblast growth factor-2; FLT-3L, Fms-related tyrosine kinase 3 ligand; CX3CL1, chemokine ligand 1, also known as fractalkine; IL-1b, interleukin-1b; IL-4, interleukin-4; IP-10, interferon gamma-induced protein 10, also known as C-X-C motif chemokine ligand 10 (CXCL10); MCP-3, Monocyte chemotactic protein-3; MIP-1b, macrophage inflammatory protein 1-beta; aSAH, aneurysmal subarachnoid hemorrhage; Statistical method: Spearman.

Correlations for all measured serum biomarkers at both measurement time points were examined. The Spearman r coefficient of correlation between all these parameters is presented as a heat-map in Figure 2 The heat-map confirmed a positive and strong correlation between IL-1b and FGF-2, CX3CL1 and MCP-3, as well as between MCP-3 and FGF-2 at T1 time point. For biomarkers measured at T2, only the correlation between MCP-3 and CX3CL1 remained strong. For more correlations see Figure 2.

**Figure 3:**
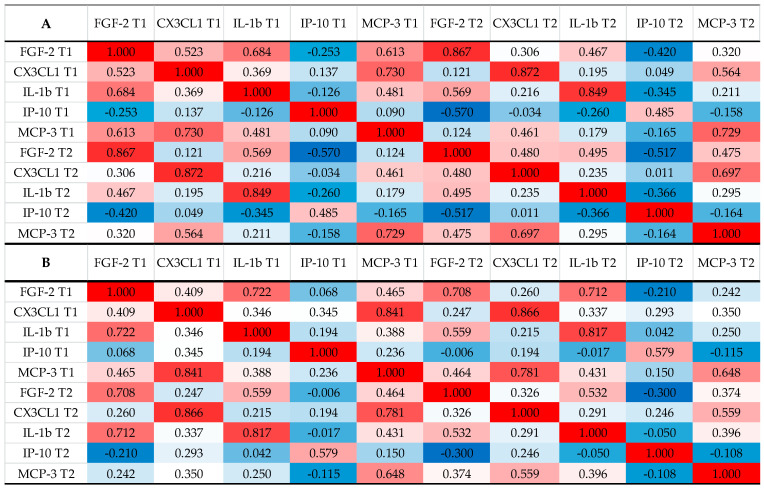
Correlation between serum biomarkers in different clinical subgroups ((**A**): favorable, *n* = 58; (**B**): unfavorable, *n* = 58). Unfavorable, mRS = 3–6 on Day 30; favorable, mRS = 0–2 on Day 30; DCI, delayed cerebral ischemia. (note: red indicates that the two parameters were positively correlated, and blue indicates that the two parameters were negatively correlated. The darker the color, the stronger the correlation). T1, serum sample at Day 1 after aSAH; T2, serum sample at Day 5–7 after aSAH. FGF-2, fibroblast growth factor-2; FLT-3L, Fms-related tyrosine kinase 3 ligand; CX3CL1, chemokine ligand 1, also known as fractalkine; IL-1b, interleukin-1b; IL-4, interleukin-4; IP-10, interferon gamma-induced protein 10, also known as C-X-C motif chemokine ligand 10 (CXCL10); MCP-3, Monocyte chemotactic protein-3; MIP-1b, macrophage inflammatory protein 1-beta; Statistical method: Spearman.

The binary logistic regression analysis identified serum Day 5–7 MCP-3 levels as an independent predictor for DCI status, Table 6. Serum level of FGF-2 showed a strong negative correlation with serum level of IP-10 in patients with favorable outcome, while this correlation disappeared in the case of the group with an unfavorable outcome (Figure 3).

## 3. Discussion

In this prospective study, we were able to show that:(i) MCP-3 and CX3CL1 levels measured at 5–7 days (T2) after aSAH are associated with the occurrence of DCI, (ii) early (Day 1) high levels of IP-10, MCP-3, and MIP-1b were correlated with Day 30 adverse outcome, and (iii) the serum level of Il-4 measured on Day 5–7 was significantly higher in TCD-positive patients than in TCD-negative ones.

CX3CL1was successfully analyzed in human CSF after aSAH with an increasing trend in concentration with a late peak at day 10 [10]. CX3CL1 expression is upregulated in intact neurons within the penumbra while both CX3CL1 and CX3CR1 expression are upregulated in infarcted brain in experimental stroke model in rats [14].This protein may be involved in the inflammatory response to traumatic brain injury (TBI), particularly in the accumulation of leukocytes in the injured parenchyma [19]. CX3CL1 may have dual functions of being neuroprotective and anti-inflammatory in a variety of hypoxic and excitotoxic in vitro and in vivo models, while proinflammatory and contributing to neuronal damage in others [20]. It has a direct effect on microglia and has the ability to induce the release of soluble factors that orchestrate a neuroprotective response [20]. CX3CL1 and its receptor are involved in a complex network of both paracrine and autocrine interactions between neurons and glia and have a role in microglia polarization [21]. In the acute phase following SAH, the microglia mainly appear to be activated into their pro-inflammatory (M1) phenotype, while the anti-inflammatory (M2) phenotype is more prevalent in the subacute and delayed phases [21]. In the early phase of ischemic stroke, the microglia initially demonstrate the M2-dominated activation which gradually changes into the M1 phenotype in peri-infarct regions. It seems that ischemic neurons lead microglial polarization more towards the M1 phenotype [22]. There is also evidence that while inhibiting inflammatory cytokines contributes to the protective activity of CX3CL1, it also reduces microglial activation, keeping these cells in a “switched off” state [23]. In rodent models, the intracerebroventricular administration of exogenous CX3CL1 provides a long-lasting neuroprotective effect against cerebral ischemia [24]. In our study, CX3CL1 levels on Day 5–7 were significantly higher in DCI patients, which also coincides with the time of microglia polarization of the M2 phenotype. The CX3CL1/CX3CR1 axis may play a protective role after SAH by attenuating microglia activation [25]; thus, the elevated levels of CX3CL1 in the late phase of aSAH may also contribute to the pathogenesis of DCI through its effect on microglia. A study by Zanier E.R. et al. suggested that CX3CL1:CX3CR1 signaling exacerbates the toxic cascades at early time-points whereas it is needed for long-term recovery in TBI [26]. Exogenous CX3CL1 reduced ischemia-induced cerebral infarct size and neurological deficits in rats and these CX3CL1-induced neuroprotective effects mediated by microglia were long lasting, being observed up to 50 days after pMCAO in rats [23].

Two possible mechanisms arise in aSAH: (i) high levels of CX3CL1 may indicate protective mechanisms; (ii) this increase is inadequate to avoid DCI. Based on our findings, a delayed elevation of CX3CL1 in patients with DCI rather suggests an overexpression of CX3CL1 as an adaptive mechanism, and not the insufficient CX3CL1 expression contributing per se to DCI development. In contrast, in patients without DCI, the level of secondary ischemic damage does not reach the necessary threshold required for the induction of CX3CL1 expression.

The serum MCP-1 concentrations correlated with vasospasm in patients with SAH, whereas the serum MCP-1 levels did not correlate with DCI; at the same time, concentrations of MCP-1 in the CSF, however, proved to be significantly higher in patients with angiographically demonstrated vasospasm [27]. In rat models, MCP-1 was found to have a significantly increased expression in the major cerebral arteries during cerebral vasospasm [28]. It was demonstrated that MCP-1 concentration measured in the CSF of SAH patients increased between day 1 and 5,peaking at day 3, followed by a gradual decrease thereafter [18]. In rat brain at twelve hours following ischemia, a marked increase of MCP-1 mRNA was observed, which was sustained in the ischemic cortex up to 5 days post-ischemic injury [29]. In humans, all MCPs have an overlapping chemoattractant activity on basophils and eosinophils, and they express strong chemoattractant features towards monocytes [30]. In our study, MCP-3 concentrations were significantly higher on Day 5–7 in patients with DCI compared with those without DCI, regardless the presence of hospital acquired infection. An increasing trend with a late peak at day 10 of MCP-3 was observed in CSF after SAH in humans [10]. This late peak reflects a more delayed activation post SAH that may indicate an involvement of MCP-3 in the healing processes or the development of late complications such as late vasospasm and DCI.

In our cohort, both Day 5–7 serum MCP-3 and CX3CL1 levels were significantly higher in DCI patients, suggesting that high MCP-3 levels indicate marked inflammatory activity, which is part of the pathogenesis of DCI. At the same time, we did not find a statistically significant difference between the serum MCP-3 and CX3CL1 levels (both measured at the T2 time point) measured in patients who developed DCI before and after T2 sampling. Based on this, it cannot be clearly determined whether the increase in the detected markers is a consequence or a cause.

In terms of functional outcome, we found that early (Day 1) high levels of IP-10, MCP-3, and MIP-1b were correlated with Day 30 adverse outcome. Lower concentration of IP-10 at 24 h after aSAH was independently associated with DCI [17] and its concentrations increased significantly during the first 5 days after SAH and may play a role in the development of delayed ischemic neurological deficits through simultaneous activation of monocytes and lymphocytes [18]. IP-10 have a transient burst of accumulation in the CNS during experimental autoimmune encephalitis (EAE), highlighting that astrocyte-derived IP-10 is a potential chemoattractant for inflammatory cells during EAE [31]. IP-10 and MCP-1 lead to accumulation of activated T cells and monocytes in the CSF compartment in the early stage of viral meningitis [32]. CSF level of iron and heme are associated with an inflammatory response (plasma levels of MIP-1b and IP-10) within the human brain after a hemorrhagic event, suggesting a causal relationship [33]. It has long been known that MCP-1, MCP-2, and MCP-3 are major attractants for human CD4+ and CD8+ T lymphocytes and monocytes [34,35]. Considering the fact that both IP-10 and MCP-3 peak only late in the course of subarachnoid hemorrhage [10], and both have a potent chemoattractant for inflammatory cells, the association of their early high levels with poor outcome, as we found in our study, suggests a prominent role of inflammation in the pathophysiology of early aSAH.

During our investigations, we found that the serum level of Il-4 measured on Day 5–7 was significantly higher in TCD-positive patients than in TCD-negative ones. TCD measurements of cerebral blood flow velocity are commonly used after aSAH to screen for vasospasm; however, their association with cerebral infarction is not well characterized and is still partially controversial [36,37,38]. In a very recent study, an early, mild, TCD-based vasospasm severity threshold had a high negative predictive value for DCI [39]. Al-Tamimi et al. observed significantly higher levels of IL-4 in CSF in patients with delayed ischemic neurological deficit, with peak-levels on Day 5 [40]. Early intracerebral injection of IL-4 potentially promotes neuro-functional recovery, probably through enhancing the activation of microglia M2 (protective) phenotype and inhibiting the activation of M1 (injurious/toxic) phenotype in patients with intracerebral hemorrhage [41]. Our study supports the assumption that patients with DCI and a putatively larger inflammatory response mount an even greater compensatory anti-inflammatory response reflected by IL-4 elevation. However, since the serum IL-4 level showed a correlation with TCD positivity and not with DCI, the increase in velocity detected with TCD in the arteries is a part of the development of DCI, but not the sole mechanism. Based on the above evidence, we can state that the significantly higher IL-4 level detected in TCD-positive patients shows the pathophysiological role of IL-4 in the development of vasospasm, which may be the basis of DCI.

On correlation analysis, although FGF-2 did not show a direct correlation with the outcome or the occurrence of DCI, a strong negative correlation was observed with IP-10 which was found to be associated with functional outcome. This negative correlation was particularly pronounced in patients with favorable outcome, but negligible in the unfavorable group. FGF-2 suppresses autophagy levels; hence, it may reduce post-SAH neuronal apoptosis, providing a neuroprotective role, at least partially, by activating the PI3K/Akt pathway [12,42]. Recently, triggering by receptor expressed on myeloid cell 2 (TREM2) was identified as regulator of both IP-10 and FGF-2 beside others. TREM2 is involved in the activation of IP-10, MIP-1a, and IL-8, while it inhibits FGF-2, and thus it plays a role in enhancing the microglial function, suggesting that therapeutic strategies that seek to activate TREM2 may not only enhance phagocytosis, but also inhibit apoptosis [43]. Taken together, this novel association between IP-10 as a marker influencing the post-aSAH outcome and FGF-2 suggests independent pathological pathways in the neuroinflammatory response after aSAH. Moreover, it also raises questions which require further studies to clarify the role of the FGF-2/FGFRs neurotrophic system in aSAH. Our study has several limitations. Biomarker samples were only taken at two time points after aSAH, which limits the precise analysis of the long term kinetics of the markers. The relatively lower number of cases also reduces the generalizability of our study. One of the reasons for this is that unfortunately, due to the medical emergency caused by COVID-19, long-term monitoring of patients in our institution was not possible in all cases due to limited access to medical personnel. This significantly limited the number of patients who could be screened. Sampling at two time points is insufficient to provide further information on whether the observed increase in markers is a consequence or a cause.

## 4. Materials and Methods

### 4.1. Study Design

This was a prospective observational study from a tertiary stroke treatment center in Pecs, Hungary. All patients ≥ 18 years of age with a newly diagnosed aSAH admitted to our hospital from November 2018 and December 2021 were offered enrollment into this study. Exclusion criteria were: traumatic SAH, pregnancy, hospital admission later than 24 h after ictus, no aneurysm treatment, absence of a signed consent form, underlying SARS-CoV-2 infection, and systemic diseases (chronic neurological disease, tumors, liver and/or renal insufficiency, and chronic lung disease). Written informed consent was obtained from each patient or their legal representative. All included patients underwent computed tomography (CT) or magnetic resonance (MR) angiography before admission and conventional cerebral angiography after admission and received treatment according to clinical treatment guidelines. Following the diagnosis of aSAH, according to our hospital standards, the aneurysm was treated endovascularly within 24 h. In all cases, the patient spentat least 12–14 days in the neurointensive care unit, so that expected complications (e.g., DCI) could be detected in time. DCI was screened by using transcranial doppler from admission in every day of hospital care. If DCI was suspected, MRI and catheter angiography were performed to confirm macrovascular vasospasm and DCI. If vasospasm was confirmed, intra-arterial nimodipine was administered.

### 4.2. Clinical Definitions

For each patient, data on demographics (age, sex) were collected. Basic comorbidities (hypertension, diabetes, and smoking) were identified. Aneurysm location and admission laboratory parameters (creatinine, C-reactive protein, neutrophile-lymphocyte ratio) were collected according to hospital records. Further, the severity of aSAH was assessed using World Federation of Neurologic Surgeons (WFNS) grade and modified Fisher scale, taking into consideration the amount of blood in the initial CT scan. The presence of mechanical ventilation, need for decompressive craniectomy, and extra ventricular or lumbar drainage were recorded. Transcranial Doppler (TCD) spasm indicated TCD positivity (TCD+) was diagnosed by daily transcranial Doppler measurements and defined as peak-value increase by >50 cm/s/24 h compared with the previous result or a mean value >120 cm/s in one of the main supply branches [44]. Angiographic vasospasm was defined as moderate-to-severe arterial narrowing on digital subtraction angiography not attributable to atherosclerosis, catheter-induced spasm, or vessel hypoplasia, as determined by a neuroradiologist [45]. We used the widespread, consensus definition of DCI [45]. The definition of infection were symptoms of infection with fever, elevated C-reactive protein and/or procalcitonin, and a positive diagnostic test such as chest X-ray or urine test. The clinical endpoints were DCI and unfavorable outcome (modified Rankin score ≥ 3) after 30 days after aSAH.

### 4.3. Sampling and Laboratory Analysis

Samples were collected from the patients at two time points: (1) 24 h after ictus (Day 1) and (2) 5–7 days after ictus and were stored at −80 °C until measurement. Serum concentrations of eotaxin (CCL-11), fibroblast growth factor-2 (FGF-2), Fms related receptor tyrosine kinase-3 ligand (FLT-3L), chemokine (C-X3-C motif) ligand-1 (CX3CL1) also known as fractalkine, interleukin-1b (Il-1b), interleukin-4 (IL-4), interferon gamma-induced protein 10 (IP-10), also known as C-X-C motif chemokine ligand 10 (CXCL10), monocyte-chemotactic protein 3 (MCP3), also known as Chemokine (C-C motif) ligand 7 (CCL7), and macrophage inflammatory protein-1b (MIP-1b) were determined using a customized MILLIPLEX Human Cytokine/Chemokine/Growth Factor Panel A multiplex assay (HCYTA-60K, Merck KGaA, Darmstadt, Germany) according to the manufacturer’s protocol. Briefly, 25 μL of each serum sample, standard and control, was added to the appropriate wells of 96-well plates provided with the kit together with 25 μL of assay buffer and 25 μL of the mixture of fluorescent-coded magnetic beads, each of which was coated with a specific capture antibody. After an overnight incubation at 2–8 °C for each analyte to be captured by the beads, and three rounds of washing, 25 μL of biotinylated detection antibody was introduced for an hour at room temperature. The reaction mixture was then incubated for 30 min with 25 μL Streptavidin–phycoerythrin conjugate, the reporter molecule, to complete the reaction on the surface of each bead. Following washing the plate three times it was run on the Luminex MAGPIX instrument (Luminex Corporation, Austin, TX, USA); each individual bead was identified and the result of its bioassay was quantified based on fluorescent reporter signals. Data were analyzed using the Belysa Immunoassay Curve Fitting Software (Merck KGaA, Darmstadt, Germany) in accordance with the manufacturer’s instructions. Samples were all processed by the same laboratory technician using the same equipment and blinded to all clinical data.

### 4.4. Statistical Analysis

SPSS 19.0 (SPSS Inc., Chicago, IL, USA) and Graph Pad Prism 9 software (GraphPad Software, San Diego, CA, USA) was used for statistical analysis of data. The categorical variables are presented as frequency and percentage. The continuous variables are presented as mean ± standard deviation or median (percentile 25–75). For comparison of data between two groups, the significances of inter-group differences were assessed using chi-square test or Fisher exact test for categorical data as well as Student t test or Mann–Whitney U test for continuous variables. Bivariate correlations were analyzed by Spearman’s correlation coefficient. Because a high number of correlations are possible among the 9 cytokines, a hard threshold of 0.50 was used to exclude correlations below this value and to focus only on strong correlations. A binary logistic regression model was used to identify independent predictors with respect to DCI status. All *p* values lower than 0.05 were considered statistically significant.

### 4.5. Ethical Considerations

The study was approved by the Hungarian Medical Research Council. All procedures were performed in accordance with the ethical guidelines of the 1975 Declaration of Helsinki. Written informed consents were received from all patients according to the guidance of Declaration of Helsinki when they participated in this study.

## 5. Conclusions

Our results demonstrated that MCP-3 and CX3CL1 may play a role in the pathophysiology of DCI and may be potential therapeutic targets in the pharmacological treatment of DCI. Considering that both markers can be promising in the prediction and treatment of DCI, further studies with a large number of cases are necessary to clearly define their role in the pathophysiology of DCI and thus open up new therapeutic horizons.

## Figures and Tables

**Table 1:** Patients characteristics. SAH, subarachnoid hemorrhage.

Number of Patients with Aneurysmal SAH, *n* = 112		
**Age**	**(years, mean ± SD)**	57 ± 13
Female	(N, %)	69 (61.6%)
Hypertension	(N, %)	49 (43.8%)
Diabetes	(N, %)	11 (9.8%)
Smoking	(N, %)	12 (10.7%)
Aneurysm location		
− Internal carotid artery	(N, %)	16 (14.3%)
− Middle cerebal artery	(N, %)	22 (19.6%)
− Anterior communicating artery	(N, %)	31 (27.7%)
− Posterior communicating artery	(N, %)	13 (11.6%)
− Anterior cerebral artery	(N, %)	14 (12.5%)
− Vertebrobasilar	(N, %)	16 (14.3%)
WFNS		
− 1	(N, %)	38 (33.9%)
− 2	(N, %)	24 (21.4%)
− 3	(N, %)	8 (7.1%)
− 4	(N, %)	14 (12.5%)
− 5	(N, %)	28 (25%)
modified Fischer grade		
− 1	(N, %)	1 (0.9%)
− 2	(N, %)	18 (16,1%)
− 3	(N, %)	57 (50.9%)
− 4	(N, %)	36 (32.1%)
Glasgow coma scale, on admission	median, IQR	13 (6–15)
Neutrophile-lymphocyte ratio, on admission	median, IQR	5.9 (4–10)
C-reactive protein, on admission	median, IQR	13 (4–61)
Creatinine, on admission	median, IQR	61 (50–72)
Extraventricular drainage	(N, %)	53 (47.3%)
Infection, CSF	(N, %)	7 (6.3%)
Infection, systemic	(N, %)	18 (16.1%)
Infection, CSF + systemic	(N, %)	5 (4.5%)
Mechanical ventilation	(N, %)	50 (44.6%)
Decompressive craniotomy	(N, %)	14 (12.5%)
Lumbal drainage	(N, %)	14 (12.5%)
Delayed cerebral ischemia	(N, %)	32 (29.1%)
Angiographic vasospasm	(N, %)	28 (28.3%)
Transcranial Doppler positivity	(N, %)	41 (41.8%)
Ischemiclesion on MRI	(N, %)	16 (15.8%)
Favorable outcome on Day 30 (mRS = 0–2)	(N, %)	58 (51.8%)
In-hospital death	(N, %)	15 (13.4%)

SAH, subarachnoid hemorrhage; WFNS, World Federation of Neurological Societies Score; MRI, magnetic resonance imaging; the categorical variables are displayed presented as frequency (%) and the continuous variables are displayed presented as mean ± standard deviation (SD) or median with interquartile range (IQR).

**Table 2:** Cytokines associated with DCI during hospitalization and functional outcome on Day 30.

	DCI during Hospitalization		mRSScore at Day 30	
	DCI − (*n* = 78 (71%)) vs. DCI + (*n* = 32 [29%])		Unfavorable Outcome (mRS ≥ 3, *n* = 54 (48.2%)) vs. Favorable Outcome (mRS ≤ 2, *n* = 58 (51.8%))	
Cytokines	Day 1	Day 5–7	Day 1	Day 5–7
Eotaxin	-	-	-	-
FGF-2	-	-	-	-
FLT-3L	-	-	-	-
CX3CL1	-	H *	-	-
IL-1b	-	-	-	-
IL-4	-	-	-	-
IP-10	-	-	H *	-
MCP-3	-	H **	H *	-
MIP-1b	-	-	H *	-
Total	0	2	3	0

DCI, delayed cerebral ischemia; mRS, modified-Rankin scale; FGF-2, fibroblast growth factor-2; FLT-3L, Fms-related tyrosine kinase 3 ligand; CX3CL1, chemokine ligand 1, also known as fractalkine; IL-1b, interleukin-1b; IL-4, interleukin-4; IP-10, interferon gamma-induced protein 10, also known as C-X-C motif chemokine ligand 10 (CXCL10); MCP-3, Monocyte chemotactic protein-3; MIP-1b, macrophage inflammatory protein 1-beta; H, high level; * *p* < 0.05; ** *p* < 0.001.

**Table 3:** Association of MCP-3 and CX3CL1 levels measured at T2 with the time of DCI detection.

	No DCI (*n*= 78)	Group A: DCI before T2 (*n* = 7)	Group B: DCI after T2 *(n* = 25)	*p*-Value (between A and B)
MCP-3 T2, pg/mL, median (IQR)	0 (0–11)	22 (8–27)	18 (0–32)	0.857
CX3CL1 T2, pg/mL, median (IQR)	83 (58–119)	116 (103–138)	106 (65–243)	0.691

T1, serum sample at Day 1 after aSAH; T2, serum sample at Day 5–7 after aSAH. DCI, delayed cerebral ischemia; MCP-3, Monocyte chemotactic protein-3; CX3CL1, chemokine ligand 1, also known as fractalkine.

**Table 4:** Comparison of clinical and biochemical characteristics between patients with and without DCI and between patients with unfavorable vs. favorable outcome (Day 30) in patients with aneurysmal subarachnoid hemorrhage.

Variable	DCI		*p*-Value	Functional Outcome at Day 30		*p*-Value
	DCI (*n* = 32)	No-DCI (*n* = 78)		Unfavorable (*n* = 54)	Favorable (*n* = 58)	
Age (years, mean ± SD)	54.8 ± 11	57.9 ± 14	0.223	61.8 ± 12	52.6 ± 12	<0.001
Female, N (%)	17 (53%)	50 (64%)	0.284	29 (53.7%)	40 (69%)	0.097
Hypertension, *n* (%)	11 (34.4%)	37 (47.4%)	0.210	28 (51.9%)	21 (36.2%)	0.095
Diabetes, *n* (%)	3 (9.4%)	8 (10.3%)	0.889	10 (18.5%)	1 (1.7%)	0.003
Smoking, *n* (%)	2 (6.3%)	10 (12.8%)	0.315	4 (7.4%)	8 (13.8%)	0.275
WFNS, median (IQR)	3 (1–5)	2 (1–4)	0.412	4 (3–5)	1 (1–2)	<0.001
modified Fischer grade, median (IQR)	3 (2–4)	3 (2–4)	1.000	4 (3–4)	2 (1–3)	<0.001
Glasgow coma scale, median (IQR)	9 (5–14)	14 (10–15)	0.02	6 (3–12)	14 (13–15)	<0.001
Neutrophile-lymphocyte ratio, median (IQR)	7 (5–10)	5 (3–11)	0.092	7 (4–12)	5.3 (3–8)	0.054
C-reactive protein, median (IQR)	24 (5–75)	9.5 (3–43)	0.104	41 (9–89)	6.8 (3–17)	<0.001
Creatinine, median (IQR)	61 (50–72)	60 (50–72)	0.744	63 (50–76)	59 (50–67)	0.122
Extraventricular drainage, *n* (%)	18 (56.3%)	33 (42.3%)	0.183	41 (75.9%)	12 (20.7%)	<0.001
Mechanical ventilation, *n* (%)	19 (59.4%)	29 (37.2%)	0.033	43 (79.6%)	7 (12.1%)	<0.001
Decompressive craniotomy, *n* (%)	8 (25%)	5 (6.4%)	0.006	11 (20.4%)	3 (5.2%)	0.015
Angiographic vasospasm, *n* (%)	27 (84.4%)	1 (1.5%)	<0.001	22 (52.4%)	6 (10.5%)	<0.001
Transcranial Doppler positivity, N (%)	30 (96.8%)	11 (16.4%)	<0.001	23 (54.8%)	18 (32.1%)	0.025
Ischemic lesion on MRI, N (%)	16 (50%)	0 (0%)	<0.001	16 (35.6%)	0 (0%)	<0.001

Favorable outcome = modified Rankin score 0–2, unfavorable = 3–6. The categorical variables are presented as frequency and percentage, and the continuous variables are presented as mean ± standard deviation or median (percentile 25–75). The significances of inter-group differences were assessed using chi-square test or Fisher exact test for categorical data as well as Student t test or Mann–Whitney U test for continuous variables. IQR, interquartile range; DCI, delayed cerebral ischemia; WFNS, World Federation of Neurological Surgeons; MRI, magnetic resonance imaging.

**Table 5:** Correlation between the occurrence of infection the appearance of DCI and biomarker values in aSAH patients.

	No Infection			Infection during Hospitalization		
	No DCI	DCI	*p*-Value	No DCI	DCI	*p*-Value
MCP-3 T2, pg/mL, median (IQR)	0 (0–11)	12 (0–32)	0.025	0 (0–8)	23 (9–27)	0.004
CX3CL1 T2, pg/mL, median (IQR)	82 (53–118)	102 (46–201)	0.221	94 (60–179)	116 (95–166)	0.152

IQR, interquartile range; DCI, delayed cerebral ischemia; MCP-3, Monocyte chemotactic protein-3; CX3CL1, chemokine ligand 1, also known as fractalkine; T2, serum sample at Day 5–7 after aSAH.

**Table 6:** Binary logistic regression model of independent predictors of DCI status after aSAH.

	B	Wald	Sig.	Exp(B)
MCP-3 T2	0.045	5.221	0.022	1.046
GCS on admission	−0.031	−0.062	0.803	0.97
Mechanical Ventilation	−0.954	0.638	0.424	0.385
Gender	−0.974	2.496	0.114	0.378
Age	−0.026	1.062	0.303	0.974
Constant	1.593	0.922	0.337	4.917

T2, sample time: Day 5–7 after aSAH; GCS, Glasgow coma scale; aSAH, aneurysmal subarachnoid hemorrhage; MCP-3, Monocyte chemotactic protein-3; DCI, delayed cerebral ischemia.

## Data Availability

All relevant data are within the manuscript.
